# Transitions in *nirS*-type denitrifier diversity, community composition, and biogeochemical activity along the Chesapeake Bay estuary

**DOI:** 10.3389/fmicb.2013.00237

**Published:** 2013-08-30

**Authors:** Christopher A. Francis, Gregory D. O'Mullan, Jeffrey C. Cornwell, Bess B. Ward

**Affiliations:** ^1^Department of Environmental Earth System Science, Stanford UniversityStanford, CA, USA; ^2^Department of Geosciences, Princeton UniversityPrinceton, NJ, USA; ^3^School of Earth and Environmental Sciences, Queens College, City University of New YorkFlushing, NY, USA; ^4^University of Maryland Center for Environmental Science, Horn Point LaboratoryCambridge, MD, USA

**Keywords:** denitrification, nitrite reductase, *nir*S, estuary

## Abstract

Chesapeake Bay, the largest estuary in North America, can be characterized as having steep and opposing gradients in salinity and dissolved inorganic nitrogen along the main axis of the Bay. In this study, the diversity of *nirS* gene fragments (encoding cytochrome *cd*_1_-type nitrite reductase), physical/chemical parameters, and benthic N_2_-fluxes were analyzed in order to determine how denitrifier communities and biogeochemical activity vary along the estuary salinity gradient. The *nir*S gene fragments were PCR-amplified, cloned, and sequenced from sediment cores collected at five stations. Sequence analysis of 96–123 *nirS* clones from each station revealed extensive overall diversity in this estuary, as well as distinct spatial structure in the *nirS* sequence distributions. Both *nirS*-based richness and community composition varied among stations, with the most dramatic shifts occurring between low-salinity (oligohaline) and moderate-salinity (mesohaline) sites. For four samples collected in April, the *nirS*-based richness, nitrate concentrations, and N_2-fluxes_ all decreased in parallel along the salinity gradient from the oligohaline northernmost station to the highest salinity (polyhaline) station near the mouth of the Bay. The vast majority of the 550 *nirS* sequences were distinct from cultivated denitrifiers, although many were closely related to environmental clones from other coastal and estuarine systems. Interestingly, 8 of the 172 OTUs identified accounted for 42% of the total *nirS* clones, implying the presence of a few dominant and many rare genotypes, which were distributed in a non-random manner along the salinity gradient of Chesapeake Bay. These data, comprising the largest dataset to investigate *nirS* clone sequence diversity from an estuarine environment, also provided information that was required for the development of *nirS* microarrays to investigate the interaction of microbial diversity, environmental gradients, and biogeochemical activity.

## Introduction

Denitrification, the dissimilatory reduction of nitrate and nitrite to gaseous products (NO, N_2_O, N_2_) under suboxic conditions, is a major biological loss term for fixed nitrogen from terrestrial and aquatic ecosystems to the atmosphere (Devol, 2008). In estuarine sediments, denitrification is capable of removing significant quantities (>50%) of nitrate from the water column, providing a sink for nitrogen, and thereby playing an important role in ameliorating the degree of eutrophication in waters subjected to external (agricultural or urban) N inputs (Seitzinger et al., 2006; reviewed by Boynton and Kemp, 2008). The anaerobic oxidation of ammonium to nitrogen gas (anammox) also contributes to the loss of fixed nitrogen in aquatic systems, particularly in suboxic water columns (Dalsgaard et al., 2003; Kuypers et al., 2003, 2005; Francis et al., 2007; Lam et al., 2009; Ward et al., 2009), but anammox is thought to be less quantitatively significant in estuaries (Risgaard-Petersen et al., 2004; Trimmer et al., 2005), including the Chesapeake Bay (Rich et al., 2008). Sedimentary denitrification is supported both by nitrate diffusing from the overlying water and by nitrate produced by nitrification within the sediment (Kemp et al., 1990; Jensen et al., 1993, 1994). These coupled processes are quantitatively important in the nitrogen budgets of estuarine and continental shelf sediments (Christensen et al., 1987; Cornwell et al., 1999). Considering the tremendous importance of denitrification in estuarine systems, it is critical to understand the distribution, diversity, and biogeochemical activity of the underlying denitrifier communities within estuaries.

Because the metabolic potential for denitrification is widespread among many phylogenetically unrelated groups, including over 50 different genera, a 16S rRNA-based approach is not generally appropriate for characterizing complex denitrifying communities. Instead, the functional genes encoding key metalloenzymes in the denitrification pathway have proven to be useful molecular markers for denitrifying organisms. In particular, nitrite reductase (NiR) catalyzes the first committed step to a gaseous product (Zumft, 1997), distinguishing true (gas-producing) denitrifiers from nitrate-respiring microbes (including those that perform dissimilatory nitrate or nitrite reduction to ammonium; DNRA). NiR occurs in two distinct forms that are structurally different but apparently functionally equivalent: NirS, containing iron (cytochrome-*cd*_1_); and NirK, containing copper (spectroscopic types I and II). Due to the critical role of nitrite reductase in the dentrification pathway, the *nirK* and *nirS* genes have been most frequently targeted for molecular diversity studies in many environments, including soils (Prieme et al., 2002; Rösch et al., 2002; Sharma et al., 2005; Smith and Ogram, 2008); groundwater (Yan et al., 2003); wastewater (Yoshie et al., 2004); suboxic water columns (Jayakumar et al., 2004, 2009; Castro-González et al., 2005; Oakley et al., 2007); and coastal and marine sediments (Braker et al., 2000, 2001; Liu et al., 2003; Santoro et al., 2006). To date, however, the molecular diversity of estuarine nitrite reductase genes has only been explored in detail within a few systems (Nogales et al., 2002; Hannig et al., 2006; Dang et al., 2009; Abell et al., 2010; Mosier and Francis, 2010). Recent studies of bacterial ammonia monooxygenase subunit A (*amoA*) genes have revealed a pattern of ammonia oxidizer diversity correlated with salinity, as well as distinct communities in freshwater and high salinity estuarine environments (Francis et al., 2003; Bernhard et al., 2005; Ward et al., 2007; Mosier and Francis, 2008). While similar patterns might be expected for the distribution of denitrification genes along the estuary, denitrifier diversity might also be related to the distribution of suboxic environments and denitrification rates, which in turn depend on the availability of key factors like organic carbon, oxygen, and nitrate.

In the present study, we explore the distribution and diversity of cytochrome *cd*_1_-type nitrite reductase (*nirS)* sequences in sediments of the Chesapeake Bay. This is the largest estuary in North America, and denitrification is a critical component of the N cycle, which is dominated by sediment N transformations. We have previously examined ammonia-oxidizing (AO) communties in these sediments (Francis et al., 2003), and the abundance and expression of key *nirS*-type genotypes at three sites in this estuary (Bulow et al., 2008). Here the fine-scale diversity, community composition, and phylogeny of *nirS* sequences at five stations were analyzed, along with *in situ* benthic N_2_-flux rates, in order to explore spatial variability in estuarine denitrifier diversity and function. The data described in this study are also significant because it represents the largest clone library-based survey of *nirS* sequence diversity in an estuary and the dataset has been used to develop a *nirS* microarray that can more efficiently investigate the interaction of microbial diversity, environmental factors, and biogeochemical activity.

## Materials and methods

### Site description

**Figure 1:**
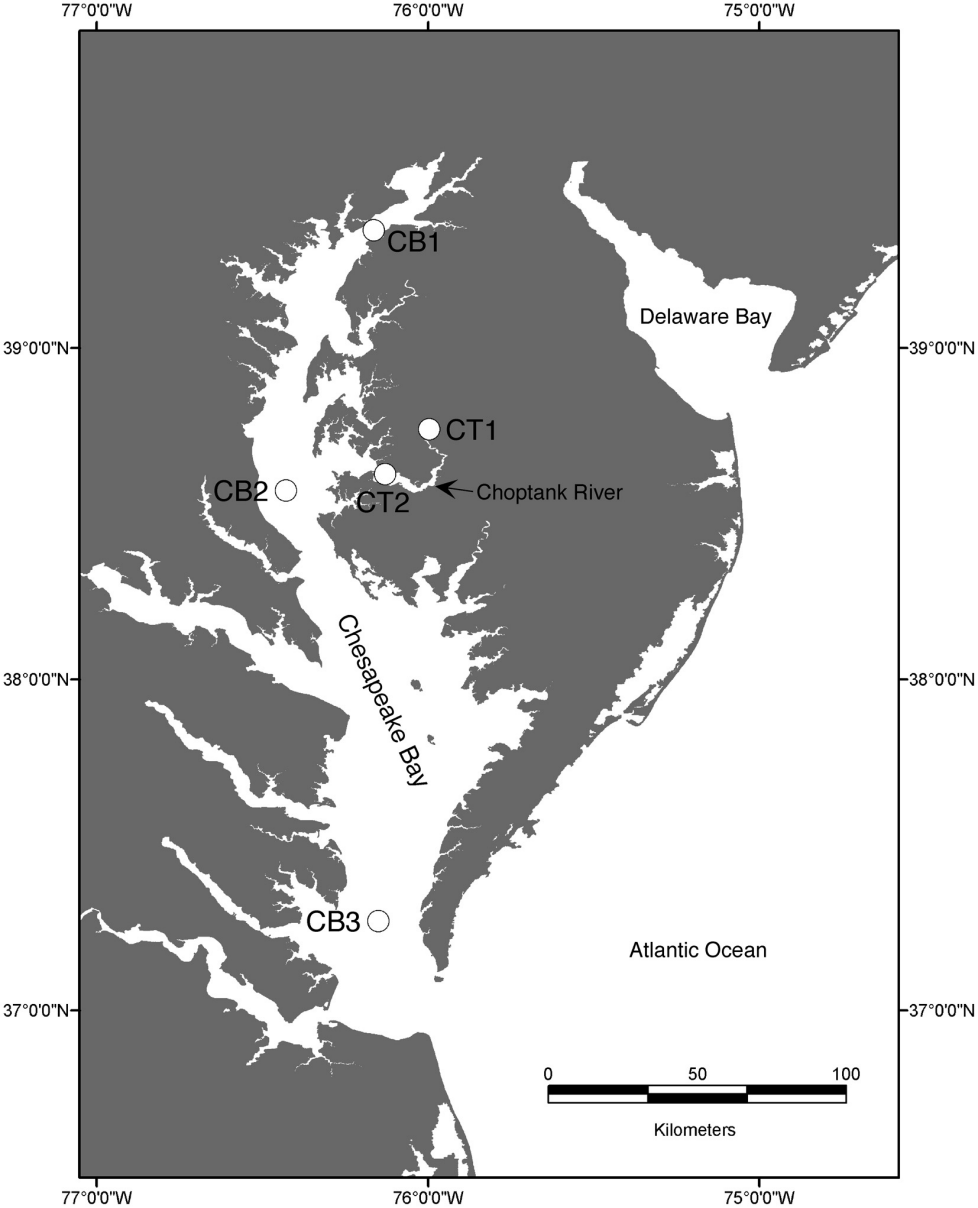
**Map of the Chesapeake Bay sampling stations**.

The Chesapeake Bay drains a watershed of 166,000 km^2^ and fills a dendritic river valley system consisting of a main channel and 7 main rivers, including the Choptank River, a subestuary that contributes roughly 1% of the total freshwater to the bay. Five stations (Figure 1) were chosen to represent the range of salinity and environmental conditions encountered along the estuarine gradient, from nearly freshwater (oligohaline; CB1, CT1) to mesohaline (CB2, CT2) to polyhaline (CB3).

### Collection and N_2_-flux analysis of intact sediment cores

Sediments were collected from upper (CT1) and lower Choptank River (CT2) stations, as well as mainstem Chesapeake Bay stations (CB1, CB2, CB3; Figure 1) using a box core sampling device deployed from either a small boat or a research vessel in April 2001 (Francis et al., 2003). Sediment samples collected in July 2000 from the upper Choptank River (CT1) were also analyzed in this study, to provide some basis for comparison with the other stations, because a subsample for molecular analysis from CT1 in April 2001 was not available. As reported previously (Francis et al., 2003), bottom water conditions measured at each sampling site are displayed in Table 1. Bottom water temperature, salinity and dissolved O_2_ were determined with a Sea-Bird CTD or a YSI 600 sonde equipped with an oxygen electrode. Nutrient concentrations were determined on using an automated analyzer (Parsons et al., 1984) on samples collected from Niskin bottles (CB1, CB2, CB3) or using a diaphragm pump (CT1, CT2).

**Table 1:** **Bottom water environmental parameters and N_2_-flux rates for Chesapeake Bay samples analyzed in this study**.

**Station**	**Sampling date**	**Water depth (m)**	**Temp (°C)**	**Salinity (psu)**	**NH^+^_4_ (μM)**	**NO^−^_3_ (μM)**	**O_2_ (μM)**	**N_2_-N flux (μmol m^−2^ h^−1^)**
CT1	July 2000	5.5	27	0.3	5	44	NA	0
CT1	April 2001	5.5	7	0.0	11	188	NA	149 ± 51
CT2	April 2001	7	8	14.5	7	22	NA	101 ± 13
CB1	April 2001	10	6.7	4.4	10	83	278	172 ± 6
CB2	April 2001	17.5	7.2	18.7	7	22	247	20 ± 24
CB3	April 2001	11	8.7	23.6	4	3	306	8 ± 13

NA, Not available.

Benthic N_2_-fluxes were measured in subcores collected from the box cores as described previously (Kana et al., 2006). For each site, three subcores in 6.35 cm i.d. acrylic core liners (~15 cm of sediment and 15 cm of overlying water) were submersed in an incubator bath of oxic bottom water from the core sampling site, and held overnight with continual aeration and circulation of the overlying water with bath water. Sediment cores and a water-only control core were capped with O-ring fitted stirring tops and incubated in the dark at *in situ* (±2°C) temperatures (see Table 1). When samples were withdrawn at various times during the incubation, replacement bottom water was supplied through a port in the stirring top, using gravity head pressure to fill vials and syringes. Solute samples were filtered using 25 mm diameter, 0.45 μm cellulose acetate syringe filters. Water for dissolved gas analysis was collected in ~7 ml ground glass test tubes that were filled through a small tube placed in the bottom of the vial to minimize gas exchange. Samples were preserved with 10 ml 50% saturated HgCl_2_ and stored at near ambient bottom water temperature until analysis.

Incubations were sampled for solutes and gases four times over a time course of 4–8 h, depending on the degree of oxygen depletion. Oxygen concentrations were occasionally monitored using an oxygen electrode early in the incubation, to determine incubation time intervals such that oxygen did not fall below 50% of air saturation by the final time point.

A quadrupole mass spectrometer with a silicone membrane inlet (Kana et al., 1994, 1998) was used for the analysis of N_2_ and O_2_ in flux samples. The N_2_:Ar ratios were corrected for any changes due to decreasing O_2_ concentrations (Kana and Weiss, 2004). Nitrate was analyzed via segmented flow analysis after Cd reduction, and ammonium was manually analyzed using the phenol hypochlorite colorimetric method (Parsons et al., 1984). Benthic N_2_ fluxes were calculated from the linear regression of the rate of change of N_2_ concentrations. At the end of the flux measurements, the cores were subsampled using cut-off 5-cc syringes. The sediment was frozen immediately in liquid nitrogen and stored on dry ice or at −80°C until DNA extraction.

### Pcr amplification and cloning of *nirS* gene fragments

DNA was extracted from replicate ~0.25 g sediment subsamples (0–0.5 cm depth interval) using the FastDNA SPIN kit for soil (MP Biomedicals), as described in Francis et al. (2003). *nirS* gene fragments (~840–890 bp) were amplified from pooled sediment DNA extracts using the PCR primers (nirS1F and nirS6R) and conditions described by Braker et al. (1998). Products were visualized by electrophoresis in 1.2% agarose gels stained with ethidium bromide. Triplicate PCR reactions were pooled, gel-purified using the QIAquick gel extraction kit (Qiagen), and cloned into the pCR2.1 vector using the TOPO-TA cloning kit (Invitrogen). Insert-containing transformants were transferred to 96-well plates containing LB broth (with 50 μg/ml kanamycin) and grown overnight at 37°C. Clones were screened directly for the presence of inserts by PCR using T7 and M13R vector primers. Sediment DNA extracts were also screened multiple times using two different *nirK* primer sets, nirK1F/nirK5R (Braker et al., 1998) as well as Cunir3/Cunir4 (Casciotti and Ward, 2001), but no consistent amplification was observed (except for the positive control DNA templates).

### Sequencing, richness and phylogenetic analysis of *nirS* sequences

Sequencing of both strands of T7/M13 PCR products was performed using ABI 310 and 3100 capillary sequencers (PE Applied Biosystems). Nucleotide sequences were assembled, edited, and aligned using Sequencher™ v.4.2 (GeneCodes Corp.), and translated using MacClade (Maddison and Maddison, 2003). Two different types of phylogenetic analysis were performed, based on nucleotide and amino acid alignments, respectively. The *nirS* nucleotide alignment (of 550 sequences) was used to define operational taxonomic units (OTUs) on the basis of DNA sequence identity. Distance matrices based on this nucleotide alignment were generated using the PAUP software package. To compare the relative *nirS* richness within each clone library, rarefaction analysis was performed. For this analysis, OTUs were defined as *nirS* sequence groups in which sequences differed by ≤5% using the furthest neighbor method in the MOTHUR program (Schloss et al., 2009).

Deduced amino acid sequences of 550 *nirS* PCR products (after removal of the primer sequences) from the Chesapeake Bay were aligned with representative database sequences (as of July 2012) using ClustalX (Thompson et al., 1997), edited in MacClade, and subjected to phylogenetic analysis. A total of 280 amino acid positions were used in the phylogenetic analysis (shorter database sequences were not included). Neighbor-joining and parsimony trees were constructed based on amino acid alignments using the PAUP software package. Bootstrap analysis was used to estimate the reliability of phylogenetic reconstructions (1000 replicates).

### Statistical analyses

Correlation analysis of environmental variables (e.g., NH^+^_4_, NO^−^_3_, and salinity) was performed in JMP (SAS Institute, 2002). Extrapolated richness [Abundance-based Coverage Estimators (ACE) and Chao1] and classical diversity (Shannon and Simpson's index) estimates were computed using MOTHUR (Schloss et al., 2009). PC-ORD software version 4.01 (McCune and Medford, 1999) was used for multivariate analyses of OTU and environmental data. OTU data were normalized for each site by dividing the number of clones per OTU by the total number of clones sequenced from the site. Environmental data were normalized by dividing the value for each variable at each site by the maximum observed value across sites. Cluster analyses (McCune and Grace, 2002), based on Sorenson distances, were performed for both OTU and environmental matrices. A Mantel Test (Smouse et al., 1986) was used to compare the significance of the observed cluster structure to the structure determined from 1000 randomizations of the matrices.

### Nucleotide sequence accession numbers

**Figure 2:**
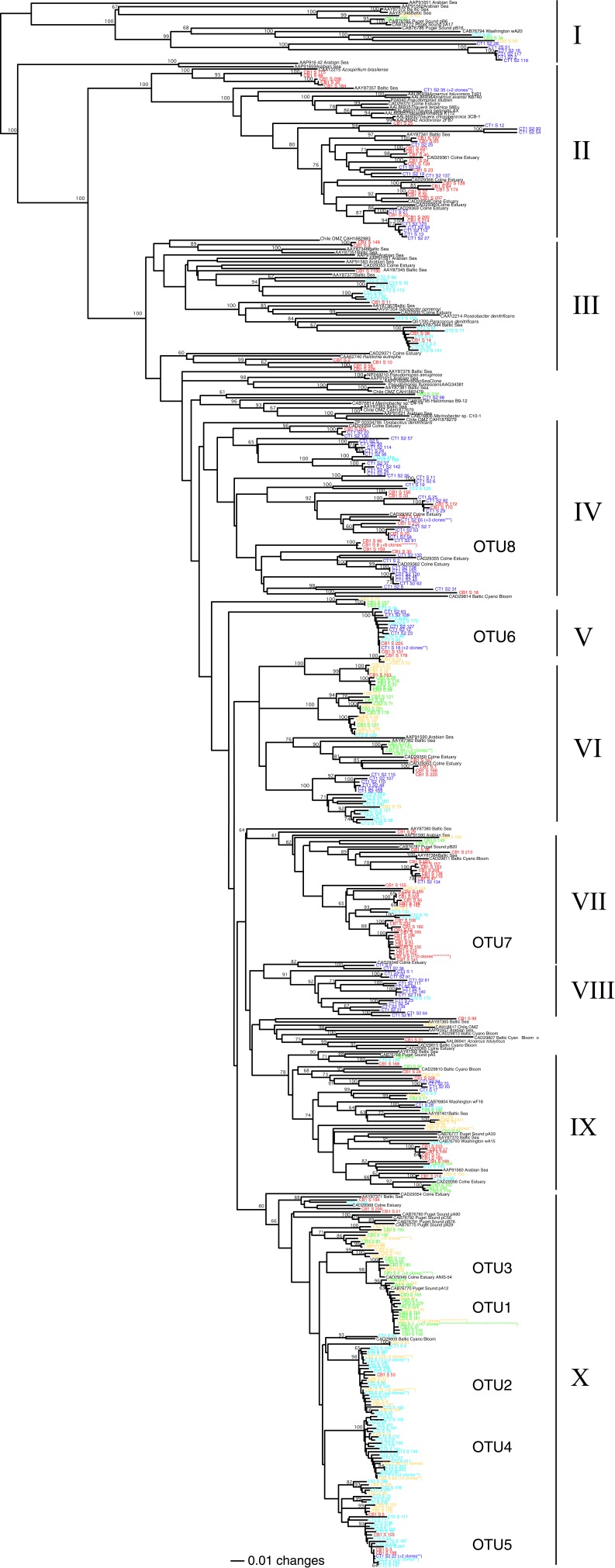
**Neighbor-joining phylogenetic tree of deduced NirS amino acid sequences (280 positions considered) from Chesapeake Bay sediments**. Bootstrap values (≥60%) are shown at the branch points. Sequences from the present study are shown in color by station (CB1, red; CB2, yellow; CB3, green; CT1, blue; CT2, aqua). Database sequences are shown in black along with the corresponding GenBank accession numbers. The number of clones identical at the amino acid level is indicated in parentheses (only for those sequences occurring >2 times within a clone library). Roman numerals refer to the ten clusters discussed in the text, all of which were also present in the parsimony tree (not shown). Clusters of NirS sequences corresponding to the 8 major nucleotide-based OTUs (defined according to 5% nucleotide sequence difference using the farthest neighbor method) are also indicated.

The GenBank accession numbers of the *nirS* sequences from cultivated denitrifiers and environmental clones used for comparison are displayed in Figure 2. The 550 *nirS* sequences reported in this study have been deposited in GenBank under accession numbers DQ675693 to DQ676242.

## Results and discussion

### Environmental gradients along the estuary

**Figure 3:**
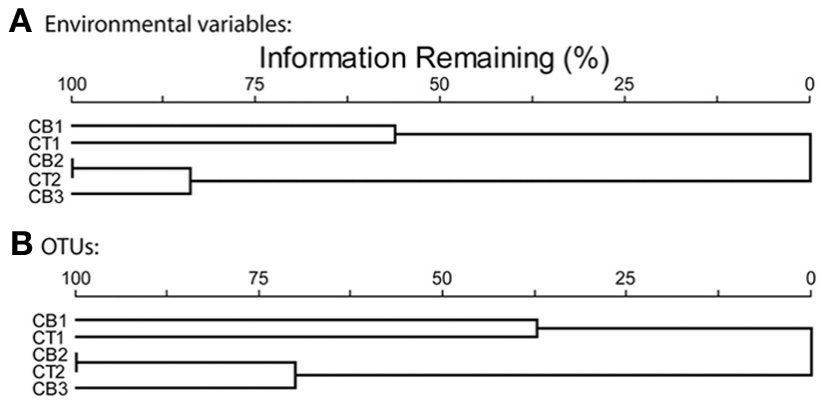
**Cluster analyses of five Chesapeake Bay sites based on relativized data using Sorenson distances. (A)** ammonium, nitrate and salinity data, **(B)** OTU distribution data, defined according to 5% nucleotide sequence difference using the farthest neighbor method.

The five Chesapeake Bay stations have been described previously in general terms (Francis et al., 2003) and the specific bottom water conditions at the time of sampling for this study are detailed in Table 1. Along the longitudinal transect from the North Bay (CB1) to South Bay (CB3) station in April 2001 (Figure 1), the salinity increased from 4.4 to 23.6 psu (Table 1). While NH^+^_4_ concentration decreased gradually from 10 to 4 μM, concentrations of NO^−^_3_—the primary electron acceptor for denitrification and generally an indicator of agricultural or urban runoff in estuarine systems—exhibited a much steeper gradient along this same transect, decreasing from 83 to 3 μM. Similar opposing gradients of salinity and inorganic nitrogen were observed from the oligohaline upper station of the Choptank River (CT1) to the mesohaline lower Choptank station (CT2) (Table 1). The overall physical/chemical conditions at the two mesohaline stations, CT2 and CB2, were quite similar, with identical levels of NO^−^_3_ and NH^+^_4_ and salinities of 14.5 and 18.7 respectively. Key differences between the environmental conditions at CT1 in July 2000 and April 2001 were temperature (27°C and 7°C, respectively) and NO^−^_3_ concentration (44 μM and 188 μM, respectively). Oxic conditions were present in the bottom waters of all stations at the time of sediment sampling. Nitrate concentration and salinity were negatively correlated (Spearman ρ = −0.93; *p* = 0.008). Cluster analysis of the sites based on normalized values of salinity, nitrate, and ammonium resulted in the formation of two distinct groups (Figure 3). The first group was comprised of CB1 and CT1 and the second group comprised of CB2, CT2, and CB3. The two most similar sites were CB2 and CT2.

### Benthic N_2_ fluxes

The N_2_-fluxes measured in sediment cores collected from five stations in April 2001 were negatively correlated to salinity (Spearman ρ = −0.90; *p* = 0.037), ranging from a high of 172 μmol N m^−2^h^−1^ at CB1 to a low of 8 μmol N m^−2^h^−1^ at CB3 (Table 1). Although this trend also generally paralleled the nitrate gradient along the Bay, the benthic N_2_-fluxes at the two mesohaline sites were quite different (CT2 rates were 5-fold greater than at CB2), despite identical (22 μM) bottom water nitrate concentrations. This difference could be due to greater coupling to nitrification at CT2, where the sediments do not experience seasonal anoxia. In contrast, the sediments at the much deeper CB2 station (18-m vs. 7-m depth at CT2) are exposed to seasonally anoxic conditions and have higher levels of pore water hydrogen sulfide (Cornwell and Sampou, 1995), which can inhibit both nitrification and denitrification (Joye and Hollibaugh, 1995). Interestingly, benthic N_2_-fluxes were undetectable at the upper Choptank River station, CT1, during July 2000, but were quite high in April 2001 (Table 1). These spatial differences, plus seasonal differences illustrated by a wide range of rates at a single site (CT1), highlight the extensive variability often associated with microbial nitrogen transformations in estuarine systems (Cowan and Boynton, 1996; Boynton and Kemp, 2008). The benthic N_2_ fluxes reported here represent the sum of both conventional denitrification and anammox. However, anammox has been shown to account for only 10-20% of the total benthic N_2_ flux at stations CB1, CT1, and CT2, (Rich et al., 2008), and was undetectable at the low-nitrate station CB3. These findings are consistent with previous studies of anammox in other estuarine systems (Risgaard-Petersen et al., 2004; Trimmer et al., 2005), and suggest that denitrification is the dominant N-removal process within Chesapeake Bay sediments.

### Analysis of *nirS* richness in Chesapeake Bay sediments

PCR amplification of *nirS* gene fragments was obtained from sediment DNA extracts from all five stations. Clone libraries were subsequently generated for each station, and 96 to 123 clones per library were completely sequenced (~840–890 bp), resulting in an overall database of 550 *nirS* sequences from the Chesapeake Bay estuary. This represents the most extensive clone library-based sequencing effort, to date, of *nirS* sequences from any system, let alone an estuary. Since *nirK* could not be reliably amplified (i.e., PCR results ranged from faint, non-specific, or multiple bands to no amplification) from all five of these sediment DNA extracts using several primer combinations (Braker et al., 1998; Casciotti and Ward, 2001), and *nirK* has been shown to be far less abundant than *nirS* in other estuarine systems (Abell et al., 2010; Mosier and Francis, 2010), we focused our efforts here on *nirS* diversity.

**Figure 4:**
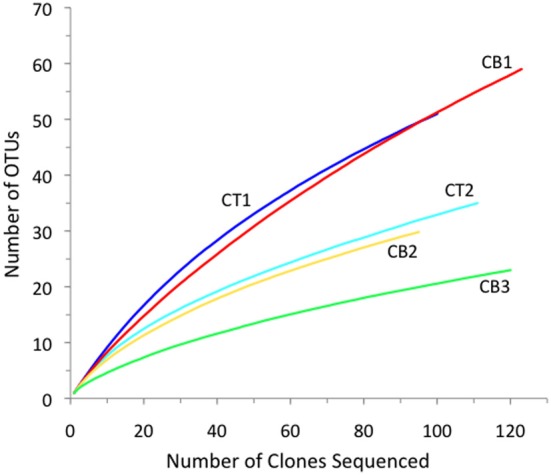
**Rarefaction curves displaying observed OTU richness versus the number of *nirS* clones sequenced from each of five Chesapeake Bay sediment samples**. OTUs were defined according to 5% nucleotide sequence difference using the furthest neighbor method.

To compare the relative *nirS*-based denitrifier richness between stations, rarefaction analysis was performed on the *nirS* sequences from using a 5% cutoff at the DNA level to define an OTU (Figure 4). Rarefaction analysis indicated the greatest *nirS* richness in the low-salinity upper Choptank River (CT1) and North Bay (CB1) libraries, and the lack of significant curvature after >95 clones suggests that that the diversity of distinct *nirS* sequences is not yet saturated in these two libraries. By far the lowest richness was observed in the South Bay (CB3) library, while intermediate levels were observed at the two mesohaline stations, CT2 and CB2. Overall, the rarefaction curves illustrate a rather striking trend among these sites spanning the estuarine salinity gradient, in which *nirS* richness decreased as salinity increased along the estuary (Figure 4 and Table 2). This trend is consistent with a previous study reporting that *nirS* diversity was inversely correlated with salinity in a wastewater treatment plant (Yoshie et al., 2004). Interestingly, no clear trends in *nirS* richness across estuarine salinity gradients were observed in Huntington Beach (Santoro et al., 2006) or San Francisco Bay (SFB; Mosier and Francis, 2010); however, both *nirS* abundance and denitrification potential activity were correlated with salinity in SFB, further highlighting the significance of this environmental factor in large North American estuaries.

**Table 2:** **Richness and diversity statistics for *nirS* clone libraries from five Chesapeake Bay sediment samples**.

**Station**	**No. of clones**	**No. of OTUs**	Unique OTUs^*^	**ACE^†^**	**Chao1^†^**	**Shannon**	**Simpson's**
CT1	100	51	44	97	95	3.69	0.02
CT2	111	35	25	126	88	2.98	0.07
CB1	123	59	45	139	116	3.54	0.05
CB2	96	30	18	82	57	2.75	0.10
CB3	120	23	17	80	46	1.79	0.35
Combined	550	172		468	360	4.24	0.04

† ACE and Chao1 are non-parametric estimators which predict the total number of OTUs in the original sample.

* OTUs detected in only 1 of the 5 Chesapeake Bay sediment samples.

The freshwater/oligohaline stations (CT1 and CB1) had the greatest total number of OTUs that were found exclusively at one site (Table 2). Interestingly, using the same OTU definition (5% cutoff), betaproteobacterial *amoA* richness was also greatest in the North Bay (CB1) but the lowest and essentially identical levels of richness were detected at the two mesohaline stations, CT2 and CB2, and intermediate levels at CT1 and CB3 (Francis et al., 2003). Thus, the relative richness/diversity of denitrifying and AO communities (based on functional genes) may be influenced differently by physical/chemical parameters, such as salinity and oxygen. It is clear that salinity has a direct, if imperfectly understood, effect on ammonia oxidizer diversity and activity (De Bie et al., 2001; Caffrey et al., 2003; Francis et al., 2003; Bernhard et al., 2005, 2007; Ward et al., 2007; Mosier and Francis, 2008); however, it is worth noting that some studies have found other factors (e.g., pH) to be important in structuring estuarine AO communities (Dang et al., 2010). Nitrate, which covaries with salinity in this system, and organic matter flux may be more important for denitrifiers. Given the limited number of samples (5) in this study, we are not able to untangle the potentially complex influence of these factors with our data; nevertheless, the pattern of changing *nirS* diversity along the salinity gradient is striking.

Although rarefaction analysis is useful for comparing the relative observed richness among clone libraries, it is not intended to predict the actual community richness (i.e., total number of OTUs) within the original samples (Hughes et al., 2001). Therefore, we also utilized several non-parametric richness estimators and diversity indices to analyze the *nirS* clone library data (Table 2). The extrapolated richness estimates (Chao1 and ACE) were generally much higher (~2–3-fold) than the observed richness within a given library. For example, the total number of observed *nirS* OTUs within our dataset (172 OTUs) represented only 37 to 48% of the number of OTUs predicted by ACE and Chao1, respectively. Overall, the predicted *nirS* richness values basically exhibited the same trend from high to low richness along the estuarine gradient that was revealed through rarefaction analysis. The classical ecological diversity indices (Shannon and Simpson's) also supported this trend.

### Analysis of OTU distributions

**Figure 5:**
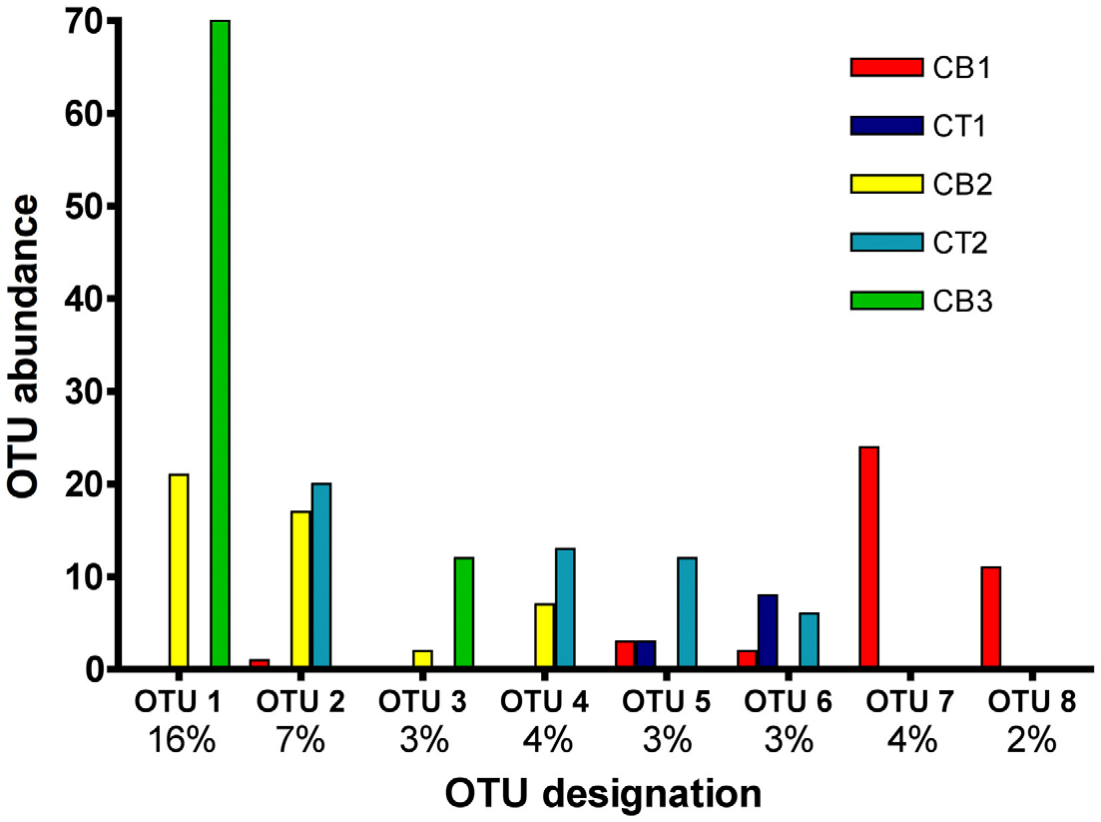
**Histogram of the eight most common OTUs from the five Chesapeake Bay *nirS* clone libraries**. OTUs were considered common if the total abundance of an OTU was ≥2% of the total number of *nirS* clones analyzed (550). The x-axis lists the OTU designation (8 of 172 OTUs are displayed), as well as the percentage of total sequences that each OTU comprises.

The number of OTUs shared between sites represents one measure of site similarity (Table 3). CT1 and CB1 had the greatest number of site-specific OTUs, while CB2 and CB3 had the greatest degree of overlap in OTU occurrence. A second measure of site similarity is the frequency of shared OTUs among sites. OTUs representing a large portion of the sequenced clones (i.e., the most abundant sequences within the clone libraries) have a large impact on this second measure of site similarity. Eight of the 172 *nirS* OTUs detected in the Chesapeake Bay accounted for 232 (42%) of the total sequences (Figure 5). Of these eight abundant OTUs, only two were unique to a particular site (OTUs 7 and 8 from CB1), while the remaining six each included sequences from two or more sites, as well as sequences from a mesohaline site. All 8 major OTUs corresponded to distinct phylogenetic clusters in the NirS amino acid tree in Figure 2. OTU1 contained the greatest number of sequences, including 71 CB3 and 20 CB2 sequences (Figure 5). The other 164 OTUs were mostly rare, 101 of which were represented by only a single *nirS* sequence (i.e., singletons).

**Table 3:** **Shared *nirS* OTUs from five Chesapeake Bay sediment samples**.

**Station**	**No. of OTUs shared with site:**				
	**CT1**	**CT2**	**CB1**	**CB2**	**CB3**
CT1	–	2	7	0	0
CT2		–	6	5	1
CB1			–	4	0
CB2				–	6
CB3					–

In order to quantify the distribution of OTUs across sites, including information from both the number of shared OTUs and the relative frequency of OTUs, a cluster analysis based on normalized OTU distribution was performed. This analysis revealed the same two station groups that had been identified in the cluster analysis of environmental data above (Figure 3). The first group was comprised of CB1 and CT1 and the second group contained CB2, CT2, and CB3 (Figure 3). The mesohaline sites, CB2 and CT2, were most similar in terms of both OTUs and environmental characteristics. A Mantel test indicated that the correspondence of OTU distribution and environmental variables was significant (*p* = 0.006). The observed clustering of environmental variables and sampling sites suggests a relationship between environmental factors and OTU distribution. However, the rather limited number of samples (5) ultimately limits our statistical power to definitively determine the impact of particular factors on the distribution of *nirS*-type denitrifier populations. While it is not always feasible (or desirable) to generate massive PCR clone libraries for the extensive number of samples necessary to perform more robust statistical approaches (e.g., non-metric multidimensional scaling), the extensive *nirS* dataset described in this study allowed the development of a microarray (Bulow et al., 2008) that can now be used to easily screen a much larger number of samples (e.g., Jayakumar et al., in press).

### Phylogenetic analysis of chesapeake BAY *nirS* sequences

In addition to comparing the relative richness and OTU distribution of *nirS* sequences, we examined the phylogenetic relationships of these sequences (Figure 2). The deduced amino acid sequences of the 550 *nirS* clones from Chesapeake Bay sediments showed only 35–85% identity to sequences of cultivated denitrifying strains. Instead, the majority of the sequences fell into phylogenetic clusters comprised primarily of Chesapeake Bay sequences and, in some cases, closely related marine and estuarine environmental clones (Figure 2).

For the purposes of this discussion, we have grouped the sequences into 10 broadly defined clusters/regions of the tree. As suggested by the extensive richness of *nirS* OTUs associated with the two oligohaline stations, CT1 and CB1, the sequences from these sites were distributed among numerous branches throughout the tree (Figure 2). However, even the less abundant sequences and OTUs exhibited substantial overlap between CB1 and CT1, as might be expected from the similarity in physical/chemical characteristics of CB1 and CT1. In fact, many sequences from these stations fell into similar regions or clusters of the phylogenetic tree, including two large clusters (II and IV) in the upper region of the tree (Figure 2) comprised almost exclusively of CT1 and CB1 sequences. Interestingly, all but one of the mRNA *nirS* clones recovered from the low-salinity, hyper-nutrified (1 mM nitrate) Hythe site, located at the head of the River Colne estuary (Nogales et al., 2002), also fell into these clusters, several of which were >95% identical to these Chesapeake sequences. The similar salinity regimes at these two geographically-distinct upper estuarine sites, despite considerably higher nitrate concentrations in the Colne estuary, support the importance of salinity (or an environmental factor that co-varies with salinity) as a key determinant in structuring denitrifying communities. Furthermore, the *nirS* sequences in Clusters II and IV apparently correspond to “low-salinity” groups of estuarine denitrifiers.

In addition to the “low-salinity” sequence types, the remaining CT1 and CB1 sequences were dispersed throughout the tree, either in discrete site-specific clusters or within clusters of sequences from other CB sites, possibly corresponding to denitrifiers that have a wide salinity tolerance. In addition to those CT1 and CB1 sequences that fell broadly into similar clusters, 13-19% of the sequences in each library were essentially identical (>99% amino acid identity) to sequences from the other site. In the absence of clone library analysis from CT1 in April 2001, comparisons between CB1 and CT1 unavoidably combine temporal and spatial variation. Even less overlap was observed in the *amoA* sequence types recovered from these two sites (Francis et al., 2003), perhaps reflecting differences in how salinity influences the composition of AO and denitrifying communities. Despite similar conditions, these upper bay and river sites experience quite different allochthonous inputs (urban vs. agricultural, respectively), which likely include microbes as well as nutrients, and these factors may interact with the physiological response to salinity.

While only three CT2 sequences fell into the two “low-salinity” clusters (II and IV), more than half (59 of 111 sequences) of the CT2 sequences fell into three distinct but closely related subclusters within cluster X (corresponding largely to OTU2, 4, and 5 from Figure 5). Cluster X contains a small number of CT1 and CB1 sequences, but is clearly dominated by sequences from mesohaline and polyhaline sites. Interestingly, there was considerable overlap between sequences from CT2 and CB2, as well as CB2 and CB3, but virtually no overlap between CT2 and CB3 (Figure 2) (also demonstrated in Figure 5 and Table 3).

The mesohaline CB2 station represents the transition zone between the North and South Bay sites, as well as the junction between the mainstem of the Bay and the Choptank River. Like the transition from CT1 to CT2, a shift in both denitrifier richness (Figure 4 and Table 2) and community composition (Figure 2) occurred between CB1 and CB2. Although the “true” (e.g., 16S rRNA-based) phylogenetic affiliations of denitrifiers cannot usually be determined based on *nirS* functional gene sequences alone, it is tempting to speculate that the shift in *nirS* sequence types from CB1 to CB2 in part reflects a major overall compositional shift in the sedimentary microbial communities between the oligohaline stations and mesohaline stations. Indeed, the transition from oligohaline to mesohaline conditions in estuarine systems is often accompanied by dramatic shifts in microbial community structure (De Bie et al., 2001), and the Chesapeake Bay estuary is no exception. Using 16S rRNA probes to enumerate the main groups of *Proteobacteria* by fluorescence *in situ* hybridization (FISH), Bouvier and Del Giorgio (2002) found consistent community shifts between the upper and lower Choptank River (CT) regions. *Betaproteobacteria* were abundant in the freshwater stations, but were rare in the lower river and the opposite pattern was observed for *Alphaproteobacteria*. The switch occurred at approximately the location of our station CT2, suggesting that a shift in the community structure of proteobacterial denitrifiers might also be expected between the two river stations, CT1 and CT2 (Taroncher-Oldenburg et al., 2003), and possibly CB1 and CB2.

Perhaps the most striking feature of the phylogenetic tree (Figure 2) is the large cluster of 83 closely-related CB3 sequences (and 23 CB2 sequences) within cluster X (corresponding to OTU1 and OTU3 in Figure 5), which share 95–100% amino acid identity to sequences of RT-PCR and PCR clones recovered from meso- to poly-haline sites within the River Colne estuary (Nogales et al., 2002) and Puget Sound (Braker et al., 2000), respectively. Within the OTU1 subcluster, 47 CB3 clones (represented by CB3-S-1) and 17 CB2 clones (represented by CB2-S-17) were 100% identical. Interestingly, these sequences also shared >99% identity with mRNA clones obtained from Narragansett Bay sediment mesocosms (Fulweiler et al., 2013). The remarkable similarity between these dominant mid- and South Chesapeake Bay sequences and sequences from multiple geographically-distinct estuaries suggests that these *nirS* genotypes may be ubiquitous in mesohaline to polyhaline (15–30 psu) sedimentary environments. Furthermore, using a *nirS* microarray, developed using sequences from this study, Bulow et al. (2008) demonstrated that sequences corresponding to the dominant CB3 *nirS* genotypes (OTU1) as well as the major CT2/CB2 sequence type (OTU2) within Cluster X were the most abundant (DNA) *and* most actively expressed (mRNA) within both CB2 and CB3 sediments. It is worth highlighting that 5 of the 8 most abundant nucleotide-based OTUs identified in the present study (Figure 5) correspond to well-defined clusters of NirS amino acid sequences within Cluster X (Figure 2), all of which are distinct from known cultivated denitrifiers. The microarray format used by Bulow et al. (2008) is capable of distinguishing *nirS* sequences that differ by 13–15% sequence identity (Taroncher-Oldenburg et al., 2003). Thus it is likely that 70-mer probes based on OTU1 and OTU2 sequences (defined based on a 5% identity cutoff) would collectively detect sequences corresponding to all 5 major OTUs within Cluster X. The microarray results verify that not only are these Cluster X sequences most abundant in Chesapeake Bay, but they also represent the most active groups.

Although the vast majority of sequences from the Chesapeake Bay were either site-specific or clustered with sequences from sites with similar physical/chemical characteristics, 5–10% of the cloned sequences from all five sites fell into one large well-supported phylogenetic cluster (IX). This cluster also included 10 clones from a number of different environments, including sediments from the River Colne estuary, Puget Sound, and Washington continental margin, as well as water column depths within the Baltic Sea and the coastal Arabian Sea oxygen minimum zone. The only cultivated member of this cluster is *Azoarcus tolulyticus*, a nitrogen-fixing betaproteobacterium that can degrade toluene under denitrifying conditions (Zhou et al., 1995; Song and Ward, 2002). This cluster is thus not only widely distributed geographically, but also among several different kinds of estuarine and marine environments.

The most divergent nitrite reductase sequences obtained in this study, sharing only 35–40% amino acid identity with the nearest cultivated denitrifier sequence, fell into Cluster I along with related sequences from several other marine and sedimentary environments. These distinct sequences represented 2% of the total 550 *nirS* sequences in this study, comprising 6% of the CT1 clones, and 1–2% of three other libraries, but were not found in the CB1 library. Although Cluster I sequences are quite distinct from most known NirS sequences, there appears to be conservation of key amino acid residues known to be critical for function. For example, Histidine 352 (*P. aeruginosa* numbering), which serves as a heme-*d*_1_ ligand in the active site of cytochrome-*cd*_1_ nitrite reductase enzymes, was conserved among Cluster I and all other sequences in this study.

## Conclusions

This study has revealed extensive and unprecedented *nirS* diversity within Chesapeake Bay estuarine sediments, with the vast majority of 550 sequences falling into numerous novel phylogenetic clusters, lineages, and OTUs, many of which may represent estuarine-specific sequence types. Both the benthic N_2_ fluxes and *nirS* gene sequences were non-randomly distributed in relation to the physical/chemical parameters observed across the five estuarine sites. While salinity was most obviously related to the benthic N_2_ fluxes and observed diversity patterns, covariation of key parameters and the limited number of sampling sites makes it difficult to definitively determine the importance of individual environmental factors in this study. A clear shift in *nirS* phylogeny and richness occurred between the freshwater and mesohaline stations, where the steepest environmental gradients were also observed. In contrast, the transition from the mesohaline mid-Bay station to the polyhaline South Bay station was less pronounced, with considerable overlap observed in *nirS* sequence types and fairly comparable richness. Sequences were not evenly distributed among the stations, however, and some dominant *nirS* genotypes (within clone libraries) were identified, especially at CB3, the most “marine” site. The eight most abundant OTUs accounted for 42% of the total sequences, consistent with the idea that *nirS*-type denitrifiers exhibit a typical “species” abundance curve, with a few very common types and many rare ones. The dominant *nirS* genotypes identified here are not obviously affiliated with known denitrifying strains, which implies that we know very little about a group of organisms that are numerically-dominant (and active in gene expression) in this system and ubiquitous in estuarine systems in general. Recent advances in high-throughput sequencing technology will undoubtedly allow future studies to more thoroughly survey the diversity of *nirS* sequences, and microarray technologies will allow a larger number of samples to be investigated so that interactions with complex environmental factors can be better understood. However, further cultivation and/or metagenomic investigations will ultimately be required to determine the phylogenetic and physiological nature of these estuarine denitrifier groups.

### Conflict of interest statement

The authors declare that the research was conducted in the absence of any commercial or financial relationships that could be construed as a potential conflict of interest.
